# Supplementary figures and images for: Coccolith mass and morphology of different Emiliania huxleyi morphotypes: A critical examination using Canary Islands material
Source: PLoS One. 2020 Mar 27;15(3):e0230569. doi: 10.1371/journal.pone.0230569 (PMC7101162; doi:10.1371/journal.pone.0230569)

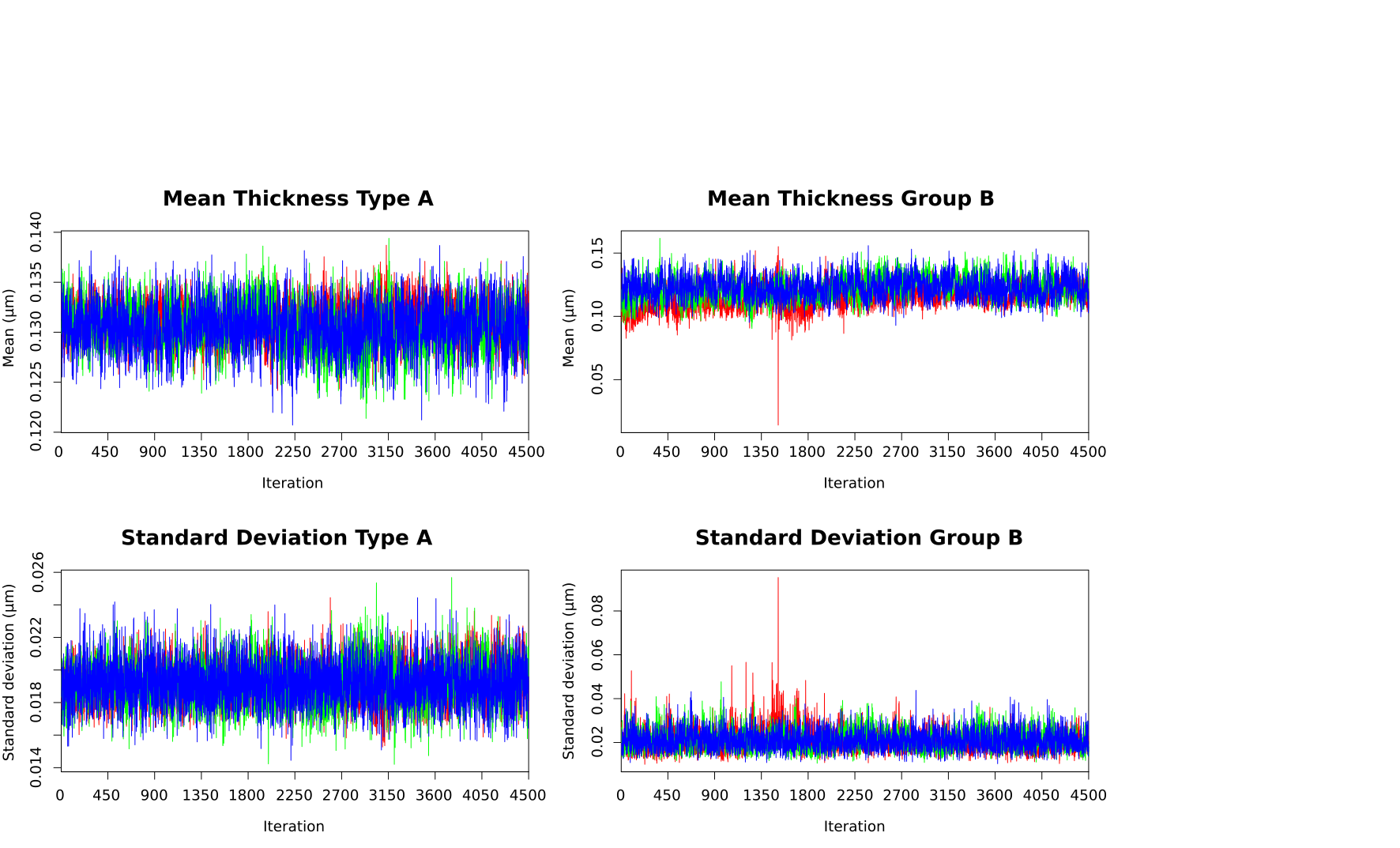

Supplement: S1 Fig — Blue, green and red lines each represent an individual chain for the sampling. (TIFF) [file pone.0230569.s001.tiff]

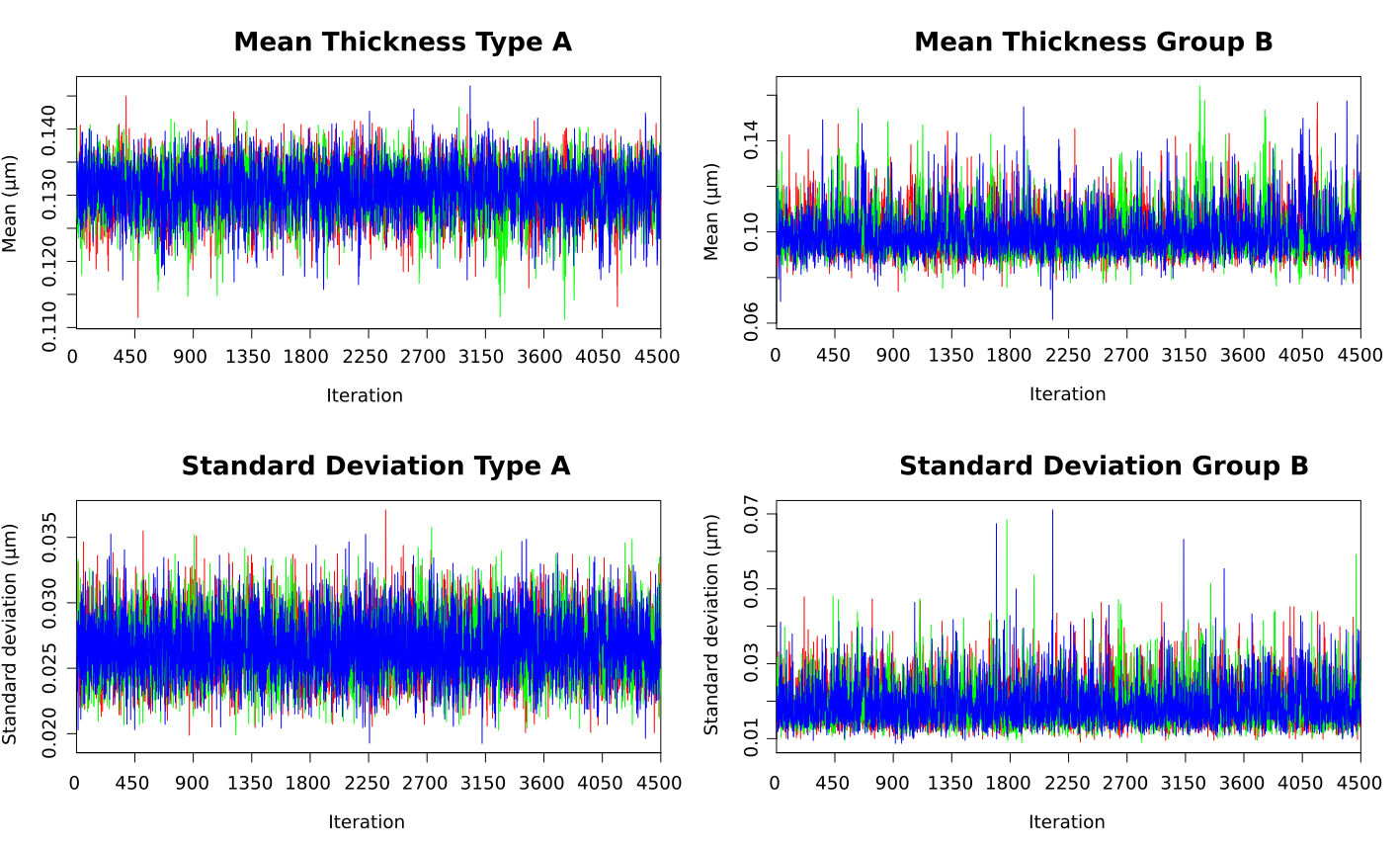

Supplement: S2 Fig — Blue, green and red lines each represent an individual chain for the sampling. (TIFF) [file pone.0230569.s002.tiff]

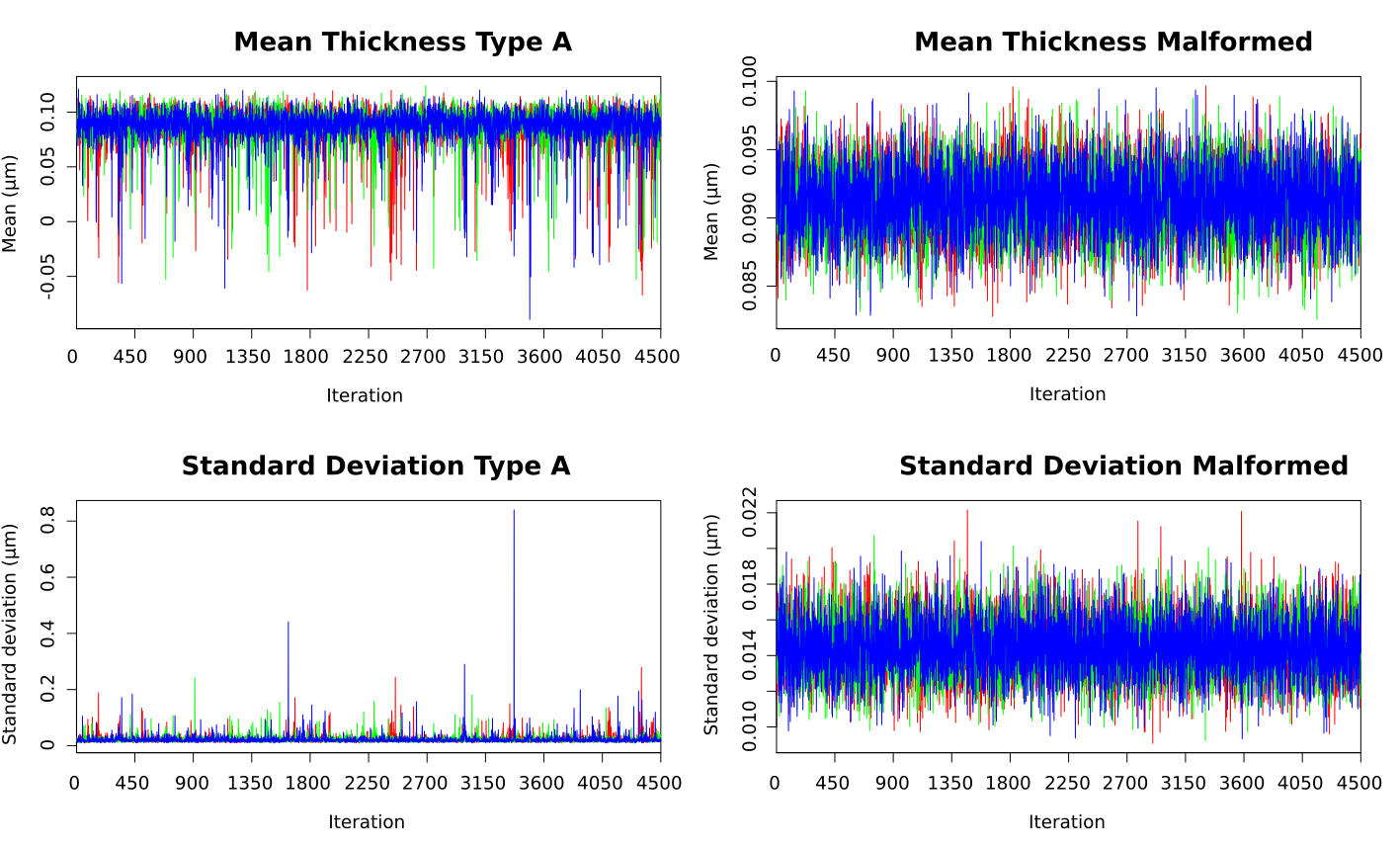

Supplement: S3 Fig — Blue, green and red lines each represent an individual chain for the sampling. (TIFF) [file pone.0230569.s003.tiff]

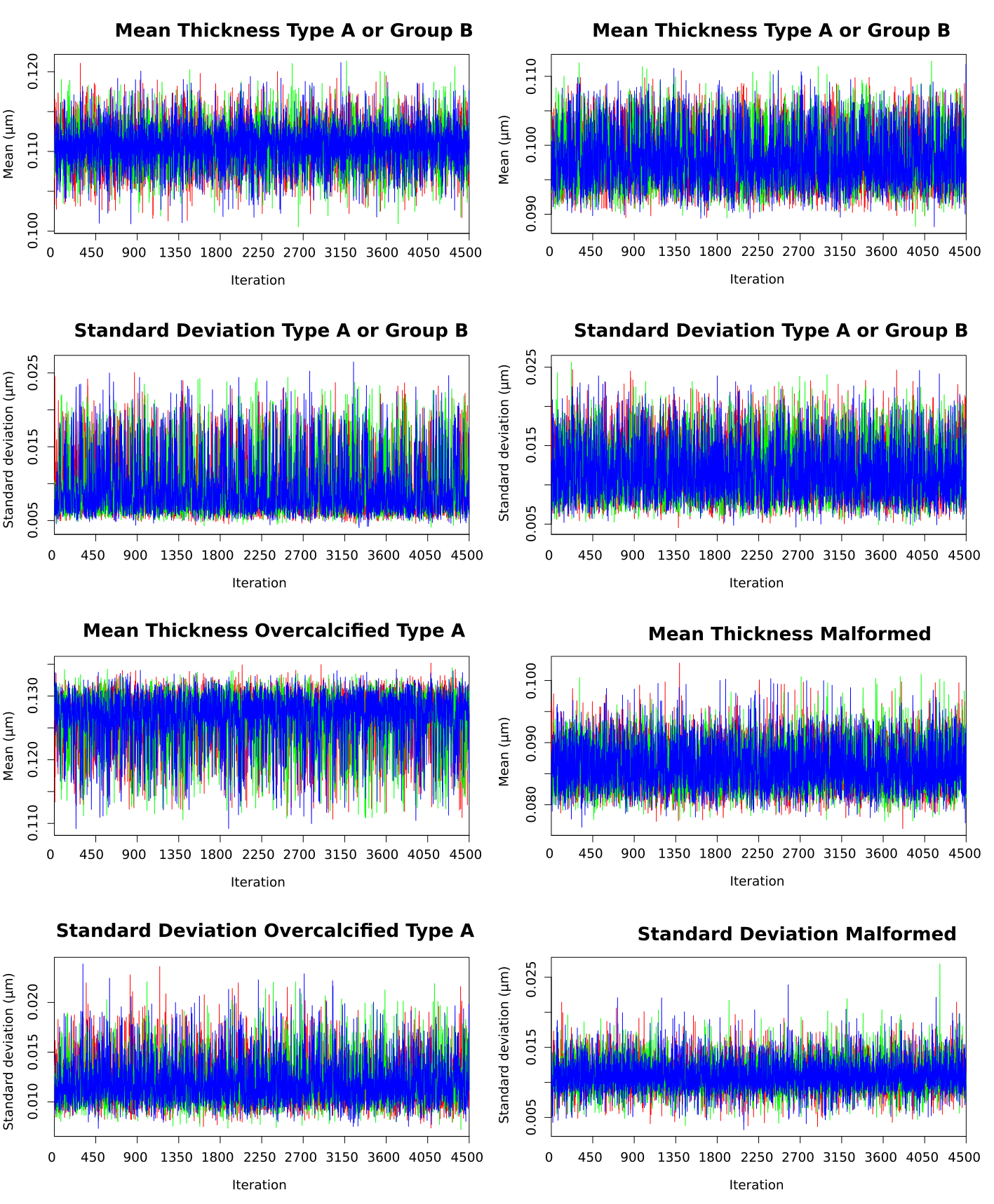

Supplement: S4 Fig — Blue, green and red lines each represent an individual chain for the sampling. (TIFF) [file pone.0230569.s004.tiff]
