# Supplementary material for: Coccolith mass and morphology of different Emiliania huxleyi morphotypes: A critical examination using Canary Islands material
Source: PLoS One. 2020 Mar 27;15(3):e0230569. doi: 10.1371/journal.pone.0230569 (PMC7101162; doi:10.1371/journal.pone.0230569)
Supplement: Attachment — Submitted filename: Response to reviewers.docx [file pone.0230569.s009.docx]

**Response to reviewers**

Reviewer #1: Overall I am happy with the authors responses, but there are a few minor things I like to see better clarified

Title: I remain unconvinced that this criticism of the use of coccolith morphotypes as a proxy for coccolith mass applies to all global Ehux populations. The work only examined Canary Island material, and hence I suggest a cautious subtitle:

"Coccolith mass and morphology of different Emiliania huxleyi morphotypes: a critical examination of Canary Island material"

**We changed the title as suggested by the reviewer.**

In the abstract, explain that CPR stands for Circular Polarizer Retardation

Final sentence of abstract: ..cannot be uniformly used as reliable indicators...

"line 362: estimated the same way
line 423: to obtain insights
line 594-95: and uncritically using coccolith morphology as a proxy...

**We changed the lines and abstract as suggested.**

Reviewer #2: The authors have thoroughly revised and improved the manuscript, including having clarified some of the methodologies, expanded the abstract and re-organized the discussion section. However, I still find some of the discussion hard to follow and need to be improved for more clarity.

The study mainly estimated the coccolith thickness and calcite mass of coccolithophore Emiliania huxleyi collected off the northwestern African coast by combining the CPR-method and MCMC analysis. The major results indicate that the coccolith length and thickness did not necessarily correlate with the characteristics of different E. huxleyi morphotypes. The calcite mass was then calculated based on the coccolith length and thickness, and the authors concluded that the morphotype appearance and relative abundance can not be used as reliable indicators of E. huxleyi calcification or calcite production.

This conclusion is contradicted with many of the previously published research. For this reason, the authors have added two paragraphs of “comparison with other studies” to explain the discrepancy between the methods used in the present study and other studies (polarized microscopic and SEM imaging methods). However, I still think the comparison is missing some important point. My major concern is that the present study mainly used the unified calcite density and simplified coccolith volume (equation 2) to generate the final conclusion, and thereby the effects of different morphotypes are ignored based on my understanding – how is this assumption valid compared to other methods? For example, Beaufort et al. (2008, biogeosciences) used the relative lightness of crystals as indicators of calcite mass.

**We agree with Reviewer Two that our results appear to contradict many previously published papers that utilised the method first published by** **Beaufort (2005). The contradictory results are not surprising because it has been demonstrated by several studies that the method published by Beaufort (2005) is flawed in many ways (see e.g. Bollmann (2013, 2014), Lochte (2014), Beaufort et al. 2014; Gonzalez-Lemos (2018). The method is not only inaccurate as it significantly overestimates coccolith weights, it also leads to** **irreproducible results because of a flawed calibration method (see publications above).**

**Having said this, the method used in our research is based on the same basic principles as reported by Beaufort (2005). It uses “**the relative lightness of crystals as indicators of calcite mass” **and** **image analysis techniques (thresholding) to detect the outline/area of a coccolith and measure the grey value of individual pixels (lightness) within the area as a proxy for coccolith thickness/mass.**

**Furthermore, using the average (****unified** **according to Reviewer Two) grey values/lightness/thickness of a coccolith to infer the mass/weight of a certain length is also a technique that Beaufort has used in many publications (e.g. Beaufort et al. (2008), Biogeosciences; Beaufort et al. (2011), Nature). Here is one excerpt from Beaufort et al. (2008) Biogeosciences page 1105 where 4 pixel/0.6µm were added to the length of coccoliths and the average (unified according to Reviewer Two) grey value/lightness/thickness were extrapolated to calculate coccolith mass/weight:**

Quote: “*The Coccolith Analyser measures the grey level of objects, their diameter and surface, and tabulates the results. There is* ***a bias of 0.6μm*** *in the measurement of the diameter of small, and dim objects, such as coccoliths. This is because we apply a minimum Grey Level threshold above background.* ***This threshold erodes 2 pixels o****f the periphery of dim objects. Each pixel is 0.15μm, and 4 pixels are eroded in total when the long diameter is measured.* ***Thus we added 0.6μm*** *to the measurement of coccolith length to compensate for this automatic loss. Another small bias in the measurements of the length of coccoliths of E. huxleyi exists, the distal shield being not detected in its entirety in cross-polarized light. There-fore the length of E. huxleyi presented here are slightly underestimated. We estimated this* ***bias to a factor of 1.25*** *by comparing measures from the Coccolith analyzer and* ***from SEM****.”*

**Therefore, we do not agree with the following concern of Reviewer Two:**

Quote “*My major concern is that the present study mainly used the unified calcite density and simplified coccolith volume (equation 2) to generate the final conclusion, and thereby the effects of different morphotypes are ignored based on my understanding – how is this assumption valid compared to other methods? For example, Beaufort et al. (2008, biogeosciences) used the relative lightness of crystals as indicators of calcite mass.”*

**In contrast to Beaufort et al. (2008), we do not extrapolate grey values/thickness to missing pixels/length. We use actual grey value/thickness measurements of coccoliths and use the average grey value of a coccolith in the LM to estimate the weight of similar sized coccoliths from SEM observations. We have not only overcome the size bias introduced by unsuitable thresholding techniques used by Beaufort (2005) by applying the Canny edge detection technique, we have also increased the taxonomic resolution significantly by using circular polarised light. In contrast to Beaufort et. al. (2008, 2011) who were only able to identify coccoliths at the family level, we were able** **to distinguish between species within the Noelaerhabdaceae.**

**In conclusion, our data quality is significantly improved compared to any publication that has used the method by Beaufort (2005). Moreover, to the best of our knowledge, there is NO study that has been able or attempted to estimate the mass of different morphotypes at sub-species level on a light microscope. Therefore, it appears to be odd that Reviewer Two takes issue with our technique, results and interpretation with reference to other light microscope studies that were not even able to distinguish between species within the Noelaerhabdaceae.**
